# Toxicogenomic Analysis of Mainstream Tobacco Smoke-Exposed Mice Reveals Repression of Plasminogen Activator Inhibitor-1 Gene in Heart

**DOI:** 10.1080/08958370802209165

**Published:** 2008-12-23

**Authors:** Sabina Halappanavar, Martin R. Stampfli, Lynn Berndt-Weis, Andrew Williams, George R. Douglas, Carole L. Yauk

**Affiliations:** Environmental Health Science and Research Bureau, Health Canada, Ottawa, Ontario; Department of Pathology and Molecular Medicine, McMaster University, Hamilton, Ontario; Environmental Health Science and Research Bureau, Health Canada, Ottawa, Ontario; Biostatistics and Epidemiology Division, Health Canada, Ottawa, Ontario, Canada; Environmental Health Science and Research Bureau, Health Canada, Ottawa, Ontario

## Abstract

Tobacco smoking is associated with cardiovascular pathology. However, the molecular mechanisms of tobacco smoke exposure that lead to initiation or exacerbation of cardiovascular disease are unclear. In this study, the effects of mainstream tobacco smoke (MTS) on global transcription in the heart were investigated. Male C57B1/CBA mice were exposed to MTS from 2 cigarettes daily, 5 days/wk for 6 or 12 wk. Mice were sacrificed immediately, or 6 wk following the last cigarette. High-density DNA microarrays were used to characterize global gene expression changes in whole heart. Fifteen genes were significantly differentially expressed following exposure to MTS. Among these genes, cytochrome P-450 1A1 (Cyp1A1) was upregulated by 12-fold, and Serpine-1 (plasminogen activator inhibitor-1, PAI-1) was downregulated by 1.7-fold. Concomitant increase in Cyp1A1 protein levels and decrease in total and active PAI-1 protein was observed in tissue extracts by Western blot assay and enzyme-linked immunosorbent assay (ELISA), respectively. Observed changes were transient and were partially reversed during break periods. Thus, gene expression profiling of heart tissue revealed a novel cardiovascular mechanism operating in response to MTS. Our results suggest a potential role for PAI-1 in MTS-induced cardiovascular pathology.

Tobacco smoking remains the second largest preventable cause of mortality and morbidity worldwide (Proctor, 2004). Exposure to tobacco smoke causes coronary disease (Price et al., 1999), atherosclerosis (Chen et al., 1995), and ischemic heart disease (Njolstad et al., 1996). The degree of this risk is proportional to the amount of smoking (Sherman, 1991). While the chemical properties of tobacco smoke are relatively well characterized, the mechanisms by which smoking leads to disease and the factors that determine susceptibility to these diseases are not well understood.

The interaction between cigarette smoke and the cardiovascular system involves complex molecular pathways that are not clearly elucidated. The use of DNA microarrays enables the simultaneous monitoring of thousands of transcripts in a single experiment and can be used to understand complex molecular responses. In the present study, we used Agilent high-density DNA microrrays to examine global transcriptional changes in heart tissue of mice exposed to mainstream tobacco smoke (MTS) for 6 or 12 wk, respectively. Our findings reveal the repression of plasminogen activator inhibitor 1 (PAI-1), a key gene involved in fibrinolysis, in the hearts of mice exposed to MTS. This finding is in contrast to established evidence demonstrating an increase in plasma PAI-1 activity and impaired fibrinolysis in cardiovascular diseases (Gils & Declerck, 2004; Hamsten et al., 1987; Juhan-Vague et al., 1987; Vaughan, 2005). We suggest a potential nonfibrinolytic role for PAI-1 following prolonged exposure to MTS.

## MATERIALS AND METHODS

### Animal Care and Husbandry

Twenty mature (8–10 wk old) male C57BL/6 × CBA F1 hybrid mice (Jackson Laboratory) were exposed to MTS (Yauk et al., 2007) using a smoke exposure system (Simani et al., 1974) adapted for mice (Hautamaki et al., 1997). Briefly, mice in individual exposure chambers were exposed to 2 cigarettes daily (1R3 reference cigarettes; Tobacco and Health Research Institute, University of Kentucky) at a rate of 0.08 L/min, 1 (20-ml) puff per 52 s, 5 days/wk for a total of 6 wk or 12 wk, including the 2-wk lead-up period (Yauk et al., 2007). Control mice were placed in restrainers only. Animals were anesthetized with isoflurane and euthanized by exsanguation. Animal procedures were carried out under the guidelines of the Canadian Council on Animal Care and procedures approved by the McMaster University Animal Research Ethics Board.

### Tissue Processing

Whole hearts were excised from the mice, snap-frozen in liquid nitrogen and stored at −80°C. At the time of experiment, frozen heart tissue was sliced randomly into two (upper and lower) halves. The upper half was used for RNA extraction. The lower half was further divided in two sections randomly for total protein extracts and microsome preparation.

### RNA Extraction and Purification

Frozen heart tissue was sliced as described earlier. Total RNA was isolated from the upper portion of the heart tissue using TRIzol reagent (Invitrogen) and purified using RNeasy Mini Kit (Qiagen). All RNA samples showed A260/280 ratios between 2.0 and 2.1. RNA integrity was determined using an Agilent 2100 Bioanalyzer (Agilent Technologies) and only high-quality RNA (28S/18S > 1.8) was used for further analysis.

### Microarray Hybridization

Individual total (2.5 μg) RNA samples of heart tissue from 40 mice (5 mice for each group, 4 treatment groups and 4 control groups) and universal reference total RNA (Stratagene) were used to synthesize double-stranded cDNA and cyanine-labeled cRNA (samples with Cyanine 5-CTP, and reference RNA with Cyanine 3-CTP, Perkin-Elmer Life Sciences) according to the manufacturer's instructions (Agilent linear amplification kits, Agilent Technologies). Cyanine-labeled cRNA targets were in vitro transcribed using T7 RNA polymerase and purified by RNeasy Mini Kit (Qiagen). Five micrograms of each labeled cRNA was hybridized to Agilent 4121A oligonucleotide microarrays (Agilent Technologies) at 60°C overnight. Arrays were washed and scanned on a ScanArray Express (Perkin-Elmer Life Sciences), and data were acquired with ImaGene 5.5 (BioDiscovery).

### Statistical Analysis of Microarray Data

A balanced blocked factorial design (Montgomery, 2001) using the date of hybridization and the date of exposure (Kerr, 2003) was used to analyze the heart data. The data were normalized using a lowess curve (Yang et al., 2002) using the SAS/STAT software, Version 8.2 of the SAS System for Windows (1999–2001, SAS Institute, Inc.). Ratio intensity plots and heat maps for the raw and normalized data were constructed using R (R Development Core Team, 2005).

Differentially expressed genes between the control and treated groups within time points were determined using the MAANOVA library (Wu et al., 2003) in R. The main effects in the model included treatment, duration of exposure, and break period, as well as all two-way interactions and the three-way interaction. This model was applied to the log_2_ of the relative intensities. The Fs statistic (Cui et al., 2005), a shrinkage estimator for the gene-specific variance components, was used to test main effects, interactions, and pairwise comparisons. The *p* values for all the statistical tests were estimated using the permutation method using residual shuffling. These *p* values were then adjusted for multiple comparisons by using the false discovery rate (FDR) approach (Benjamini, 1995).

The group means for the fold change calculation were based on the adjusted relative intensity for each gene where the estimated day of hybridization and date of exposure effects using parameter estimates from the analysis of variance (ANOVA) model were subtracted from the normalized ratio.

### Real-Time Reverse-Transcription Polymerase Chain Reaction (RT-PCR)

Primers were designed using Beacon design 2.0 (Premier BioSoft International). About 2.5 μg of total RNA per sample was reverse transcribed and quantitative polymerase chain reaction (PCR) was performed in duplicate with an iCycler IQ real-time detection system (Bio-Rad) as described in Dong et al. (2005). The values of the threshold cycle were averaged. Gene expression levels were normalized to the ubiquitin gene. PCR efficiency was examined using the standard curve for each gene. Primer specificity was assured by the melting curve for each gene. A *t*-test was used for statistical evaluation. Primer pairs used were: PAI-1 forward 5′CCCACACAGCCCATCAGG 3′, PAI-1 reverse 5′ CCGAGGACACGCCATAGG 3′, Ubc forward 5′ACCTTCCTCACCACAGTATC 3′, Ubc reverse 5′CCATCACACCCAAGAACAAG 3′, Cyr61 forward 5′GTTCCGATGCGAAGATGG 3′, Cyr61 reverse 5′CTTGTGGATGTCATTGAATAGG 3′ Cyp1A1 forward 5′ATTCCTGTCCTCCGTTACC 3′, Cyp1A1 reverse 5′AGGCTGTCTGTGATGTCC 3′.

### Preparation of Microsomes

A section of the heart was taken from five individual mice (*n* = 5/treatment group). These heart sections were pooled into one sample. Approximately 1.0 g heart was homogenized in 2.5 ml ice-cold homogenization buffer containing 0.05 *M* Tris and 1.15% KCl, pH 7.4, using an ice-cold grinding vessel (size C, Thomas Scientific). The heart homogenates were centrifuged at 10,000 × g (9000 rpm) for 20 min at 4°C. Supernatant was then centrifuged 176,000 × g (44,000 rpm) for 1 h (rotor Ty 70.1, Beckman Ultracentrifuge). The supernatant was decanted, and 0.25 *M* sucrose was added to the microsomal pellet. The pellet was dispersed by brief sonication and was quantified for total protein content using a Bradford protein assay kit (Bio-Rad).

### Preparation of Tissue Protein Extracts and Western Blotting

A portion of the frozen heart was homogenized in lysis buffer (5 *M* HEPES, pH 7.5, 5 *M* NaCl, 10% glycerol, 1% Triton X-100, 2 *M* EGTA, 1 *M* MgCl_2_, 0.5 *M* NaF, 0.2 *M* sodium pyrophosphate, and protease inhibitor cocktail tablets [Roche Applied Science]), centrifuged, and the supernatant was quantified for protein content using a Bradford protein assay kit (Bio-Rad). Approximately 200 μg of total protein was extracted from each individual mouse (*n* = 5/treatment group) from one treatment group, and subsequently pooled to make one sample. The protein content of each pooled sample was quantified again using a Bradford protein assay kit.

For Western blotting, 30 μg total protein (pooled) or 50 μg microsomal extract was immunoblotted on 8–12% sodium dodecyl sulfate polyacrylamide gel electrophoresis (SDS-PAGE) gels and analyzed using antibodies against PAI-1, Cyp1A1 (Santa Cruz Biotechnologies), and tPA (American Diagnostica). Signals were detected by ECL Plus (GE Health Sciences).

### Total PAI-1 Antigen and Activity Assay

Murine total PAI-1 antigen and activity assay kits (Innovation Research, Inc.) were used to analyze total and active PAI-1 protein present in heart tissue extracts. All incubations were carried out at room temperature for 30 min with agitation at 300 rpm. Fifty micrograms of pooled protein extracts for total and 100 μg for active PAI-1 determination in triplicates along with known amounts of PAI-1 standards in duplicates were assayed on a 96-well plate coated with capture antibody or urokinase, respectively. Unbound PAI-1 was washed and the plate was reacted first with anti PAI-1 primary antibody followed by horseradish secondary antibody. Wells were then reacted with TMB substrate solution for 7 min and reactions were quenched with the addition of 1 *N* H_2_SO_4_. The absorbance was measured at 450 nm on a microtiter plate spectrophotometer.

### Tissue Plasminogen Activator (tPA) Activity Assay

Mouse tissue plasminogen activator (tPA) activity assay kit (Innovation Research, Inc.) was used to quantify the active mouse tPA in the tissue extracts of heart. All the reactions were carried out at ambient temperature on a shaker at 300 rpm for 30 min. In brief, 96-well immulon strip plates coated with Avidin were reacted with biotinylated human PAI-1. A series of known concentrations of tPA activity standards in duplicates and 100 μg pooled total protein extracts in triplicates were added to the wells. Wells were incubated with primary anti murine-tPA antibody, horseradish peroxidase conjugate secondary antibody, and finally with TMB substrate. The reaction was quenched by the addition of 1 *N* H_2_SO_4_. Final absorbance was read at 450 nm 5 min after the addition of H_2_SO_4_.

## RESULTS

### Global Transcriptional Profiling

Complete DNA microarray data are available in ArrayExpress (http://www.ebi.ac.uk/microarray-as/aer/?#ae-main, accession number E-MEXP-1553). Approximately 85% of the 22,000 transcripts on the array were expressed (where expressed is defined as at least 4 out of 5 samples with signal intensities above background in at least 1 experimental condition). Differentially expressed genes were identified using MAANOVA; values were considered significantly different from control values when the FDR adjusted *p* value was less than .05.

Differential gene expression was identified for 15 genes in the smoke-exposed group (Table 1; 4 upregulated and 11 down-regulated by at least 1.2- to 1.5-fold compared to sham controls). The largest change was observed in Cyp1A1, with 12-fold and 8-fold increases in gene expression at 6 and 12 wk, respectively. Serpine1 (PAI-1) and Cyr61 were downregulated with 1.7- and 1.9-fold repression, respectively. Others include suppression of Lpin3, a candidate gene for human lipodystrophy (Peterfy et al., 2001); Kpna2 (Umegaki et al., 2007), an importin; and AKAP12, a tumor suppressor (Flotho et al., 2007). However, changes in gene expression were transient and were partially reversed when smoking was discontinued (Table 1, columns B and D).

**TABLE 1:** List of genes up or down regulated in the treatment groups

Description (*Mus musculus* mRNA)	Fold change			
	1	2	3	4
Cytochrome P-450, family 1, subfamily a, polypeptide 1 (Cyp1a1)	11.7	1.3	8.2	−1.0
Cysteine-rich protein 61 (Cyr61)	−1.9	−1.3	−1.1	−1.4
RIKEN cDNA 1300002F13 gene (1300002F13Rik)	−1.8	−1.4	−1.0	−1.3
H2A histone family, member Y (H2afy)	1.8	−1.2	1.1	1.0
Serine (or cysteine) proteinase inhibitor, clade E, member 1 (Serpine1)	−1.7	−1.5	−1.3	−1.5
DNA segment, Chr 4, expressed (D4Wsu53e)	1.6	1.1	1.2	1.1
Basic helix–loop–helix domain containing, class B2 (Bhlhb2)	−1.5	−1.2	−1.1	−1.2
Karyopherin (importin) alpha 2 (Kpna2)	1.5	1.1	1.0	1.0
Small proline-rich protein 1A (Sprr1a)	−1.5	1.2	−1.0	−1.2
Lipin 3 (Lpin3)	−1.4	1.2	−1.0	−1.1
Colony-stimulating factor 1 (macrophage) (Csf1)	−1.4	1.0	−1.1	−1.0
Unknown	−1.3	1.1	1.0	−1.1
A kinase (PRKA) anchor protein (gravin) 12 (Akap12)	−1.3	1.0	−1.0	−1.1
Carbamoyl-phosphate synthetase 1	−1.1	−1.3	1.3	1.3
RIKEN cDNA 4632404H22 gene (4632404H22Rik)	−1.2	−1.2	1.3	−1.1

*Note*. Fold induction over matched controls for treatment groups: 1, 6 wk smoke; 2, 6 wk smoke followed by 6 wk break; 3, 12 wk smoke; 4, 12 wk smoke followed by 6 wk break. Negative values indicate downregulated genes.

**FIG. 1:**
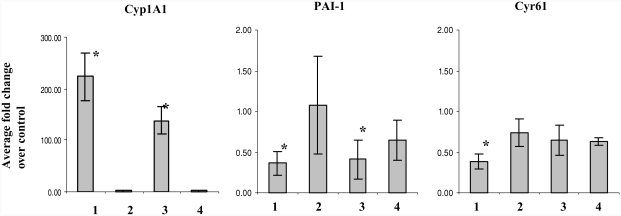
Validation of microarray results. Data are presented as fold change over control values (*n* = 5 mice/group, ± SEM): 1, 6 wk smoke; 2, 6 wk smoke + 6 wk break; 3, 12 wk smoke; and 4, 12 wk smoke + 6 wk break. Asterisk indicates significant at *p* < .05, t-test conducted on RT-PCR values.

Real time RT-PCR was performed to measure the expression levels of select genes on the same RNA samples used for microarray analysis (Figure 1). Results confirmed the upregulation of Cyp1A1, and downregulation of PAI-1 and Cyr61 in 6- and 12-wk smoke-exposed samples. The observed fold changes by real-time RT-PCR were much higher than those measured by DNA microarrays (Wang et al., 2006). In keeping with the microarray results, changes were partially reversed following smoking cessation. Gene expression changes for these genes were also confirmed by RT-PCR in heart tissues of female mice identically exposed to MTS (data not shown).

### Increased Cyp1A1 Protein Levels in Heart Microsomes

**FIG. 2:**
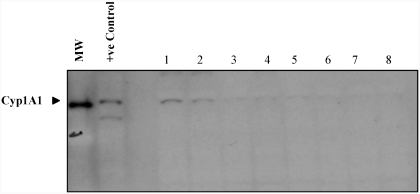
Cyp1A1 protein abundance in heart microsome extract (*n* = 5). MW: molecular weight marker; +ve control: BaP-treated lung microsome extract. Lanes 1–4: treatment groups, 6 wk smoke, 12 wk smoke, 6 wk smoke + 6 wk break, and 12 wk smoke + 6 wk break; lanes 5–8: matched controls.

Benzo[a]pyrene (BaP), a carcinogenic polycyclic aromatic hydrocarbon (PAH) found in tobacco smoke, is metabolically activated by genes in the cytochrome P-450 family. It is predicted that if BaP is metabolized in the vessel wall of the heart tissue, the initial event should be the induction of Cyp1A1 (the main form of the Cyp genes responsible for BaP metabolism in the heart) (Thirman et al., 1994). After exposure to 6 and 12 wk of MTS, heart microsomes showed distinct immunoreactive bands for Cyp1A1 (Figure 2). Similar to transcriptional induction, Cyp1A1 protein synthesis in smoke-exposed animals was highly induced compared to their matched controls, and protein levels returned to normal following the break periods.

### PAI-1 Protein Abundance and Activity

**FIG. 3:**
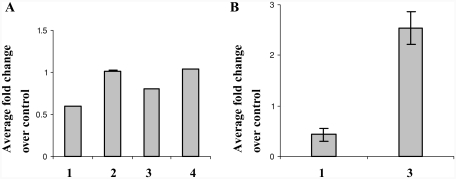
(A) Total immunoreactive PAI-1 in heart tissue extracts. (B) PAI-1 activity in heart tissue extracts. Data represent fold change over matched controls (*n* = 5, ± SEM): 1, 6 wk smoke; 2, 6 wk smoke + 6 wk break; 3, 12 wk smoke; 4, 12 wk + smoke 6 wk break.

Increased plasma levels of PAI-1 expression have routinely been found in many cardiovascular diseases (Dellas & Loskutoff, 2005). The present study is the first to show in vivo repression of PAI-1 in response to toxicant exposure, such as MTS. In order to verify that changes in mRNA levels of PAI-1 result in inhibition or decrease in its protein levels in heart, known amounts of cellular protein from whole heart tissue extract were assayed by Western blot as well as by enzyme-linked immunosorbent assay (ELISA). Reduction in total PAI-1 protein was not evident by Western blotting (data not shown). However, significant reduction (40%) in total PAI-1 protein was observed following 6 wk of MTS exposure using the total PAI-1 antigen assay (Figure 3A). Although PAI-1 levels (mRNA expression) showed marginal recovery after 12 wk of MTS exposure, total protein was still 20% less than the controls. PAI-1 levels returned to normal during the break periods.

**FIG. 4:**
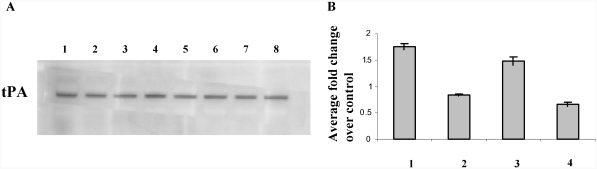
(A) Western blot of immunoreactive tPA in heart tissue extracts. Lanes 1, 3, 5, and 7 represent 6 wk smoke, 6 wk smoke + 6 wk break, 12 wk smoke, and 12 wk smoke + 6 wk smoke. Lanes 2, 4, 6, and 8 represent matched controls. (B) Total tPA activity present in heart tissue extracts. Data shown as fold change over matched controls:1, 6 wk smoke; 2, 6 wk smoke + 6 wk break; 3, 12 wk smoke; 4, 12 wk smoke + 6 wk break.

Total active PAI-1 was also measured in whole tissue extracts. Consistent with our protein analysis, at 6 wk after MTS exposure, active PAI-1 levels were less than 0.5-fold of their matched controls. Interestingly, levels of active PAI-1 protein increased 2-fold after 12 wk of exposure (Figure 3B). As mRNA expression and total protein levels of PAI-1 during the break periods did not change, we did not measure the active levels of PAI-1 in these samples. To investigate additional effects of PAI-1 downregulation, we examined total cellular tPA levels. Transcriptional repression of PAI-1 in response to MTS exposure did not affect total tPA protein levels (Figure 4A). We also quantified the total active tPA in heart tissues using a Murine tPA activity immunoassay. Although we saw a corresponding change in total active tPA (Figure 4B) with decreasing PAI-1 levels at 6 and 12 wk (Figure 3A) following MTS exposure, results were not statistically significant.

## DISCUSSION

Global analysis of gene expression in the hearts of mice exposed to MTS revealed surprisingly few genes exhibiting differential expression. Upregulation of Cyp1A1 confirmed that chemical constituents of the tobacco smoke reached the heart, and that changes in transcripts could be measured (Figure 1). Fifteen genes were differentially expressed as a result of the exposure, of which 3 (cytochrome P-450 1A1 subfamily a, polypeptide 1, cysteine-rich protein 61, and serine [or cysteine] proteinase inhibitor, clade E, member 1 [PAI-1]) were responsive at both the 6- and 12-wk time points (Table 1). The responsive genes were found to be involved in pathways related to xenobiotic metabolism and fibrinolysis. There is a strong relationship between cigarette smoking, fibrinolytic mechanisms, and elevated risk for cardiovascular events. Components of the fibrinolysis pathway—endothelium, platelets, and fibrinogen—are influenced by active smoking. Smoking causes endothelial dysfunction and impairs acute endogenous fibrinolytic capacity, a mechanism by which cigarette smoking can result in arterial thrombosis and myocardial infarction (MI) (Newby et al., 1999).

Intriguingly, PAI-1, a key inhibitor of tissue type and urokinase plasminogen activator in plasma, was downregulated in the mice exposed to MTS at both time points. PAI-1 is an acute-phase response gene and its levels in plasma are tightly regulated by local cellular environment (Kluft et al., 1985; Dellas & Loskutoff, 2005; Sawdey & Loskutoff, 1991). High levels of circulating PAI-1 contribute to the development of MI, coronary artery disease, and pathologic thrombosis (Dellas & Loskutoff, 2005; Gils & Declerck, 2004; Hamsten et al., 1987; Juhan-Vague et al., 1987; Vaughan, 2005), worsen the prognosis of patients suffering from MI, and are routinely observed in atherosclerotic plaques, macrophages, and endothelial cells of people diagnosed with cardiac diseases (Schneiderman et al., 1992). Thus, the relationship of increased plasma PAI-1 activity and cardiovascular disease incidence is well documented. However, a direct causal relationship between increased PAI-1 expression and a known cardiac disease is yet to be established. Moreover, PAI-1 knockout mice are phenotypically normal, although they are more prone to bleeding when challenged (Carmeliet et al., 1993a, 1993b) . Deficiency or total absence of PAI-1 expression in humans is associated with mild atypical bleeding diathesis and no apparent disease state (Agren et al., 2007; Santamaria et al., 2007). These studies raise important questions about the implications of altered PAI-1 expression in these disease models.

Our finding of downregulation of PAI-1 mRNA and protein synthesis in heart tissue of mice exposed to 6 and 12 wk of MTS is consistent with previous work on plasma PAI-1 levels in humans examining acute effects of smoking in chronic smokers and nonsmokers. The authors found decreased plasma levels of PAI-1 in chronic smokers compared to nonsmokers within 30 min of both groups smoking 2 cigarettes (Ozdemir et al., 1992). They suggested that platelets and endothelial cells of nonsmokers are more sensitive to acute smoking than those of chronic smokers, and that chronic smoking causes receptor downregulation or exhaustion of the platelets and endothelial cells (Ozdemir et al., 1992). Our results demonstrate that the transcriptional down-regulation of PAI-1 mRNA leads to decreased protein synthesis in whole heart tissue in mice. This finding, likely attributed to endothelial cell response, is a potential mechanism for the observed decrease in plasma PAI-1 levels measured by Ozdemir et al. (1992) in chronic smokers. Total cellular PAI-1 is much lower than secreted levels. It is the free circulating PAI-1 in plasma that is capable of binding tPA and inactivating fibrinolysis. The dramatic decrease in total protein synthesized (Figure 3A) at 6 wk following MTS was accompanied and further validated by observed differences in active PAI-1 levels (Figure 3B). Although Furuyama et al. (2006) noted that in RHMVE cells exposed to diesel exhaust particles, only secreted PAI-1, not total cellular PAI-1, levels decreased with decreasing mRNA (Furuyama et al., 2006), we did see a fairly significant change in active PAI-1 levels in heart tissue extracts. The discrepancy could be due to differences in the type of cells and chemicals used, or as a result of differences in response of cultured monolayer of cells compared to whole heart tissue. Future work will investigate PAI-1 levels in plasma.

Growth factors, cytokines, and hormones involved in inflammation and tissue remodeling induce PAI-1 expression (Loskutoff et al., 1989). Transforming growth factor (TGF) β1 is a major regulator of PAI-1 in both in vivo and in vitro models (Dong et al., 1996). Oxidative stress (Furuyama et al., 2006) and MAPK signaling pathways (Pasten et al., 2007) are shown to downregulate PAI-1 mRNA expression in vitro. Our microarray results demonstrate downregulation of KPNA2 (Table 1), an importin regulated by TGFβ1 pathway (Umegaki et al., 2007). RT-PCR results show significant downregulation of an acute response gene JunB mRNA, a downstream target of TGFβ1, and a small decrease in cytokine TGFβ1, its receptor TGFR2, following 6 wk of exposure to MTS (data not shown). Therefore, we speculate overall shutdown of acute response to MTS in heart at 6 wk following exposure to MTS.

Interestingly, we also found a small increase in PAI-1 mRNA expression (relative to 6 wk) and protein levels at 12 wk after MTS exposure (Figures 1 and 3A). An increase in blood serum and bronchoalveolar lavage fluid counts for total cell number and mononuclear cells was also observed, accompanied by an increase in interleukin (IL)-6 mRNA and protein expression in lungs of the 12-wk MTS animals (unpublished data). These data demonstrate potential initiation of inflammatory response with continuing exposure to MTS. Apart from its role in stabilizing a fibrin clot, PAI-1 is also implicated in inflammation, wound healing, and tissue repair. Vascular events including inflammation and tissue injury increase with chronic smoking and thus may necessitate reversal of PAI-1 expression. However, altered PAI-1 gene expression or protein synthesis resulted in subtle and nonsignificant changes in overall tPA levels and its activity (Figure 4).

Thus, time-dependent changes in PAI-1 expression and reversal of its repression during quit periods suggest tight regulation of PAI-1 expression and suggest separate functions for PAI-1 in response to different MTS exposure scenarios. Studies have shown that downregulation of PAI-1 reduces cardiomyocyte apoptosis after acute MI (Xiang et al., 2005). Red wine polyphenols offer cardioprotection associated with decreasing PAI-1 expression in human coronary artery endothelial cells (Pasten et al., 2007). We hypothesize that transient PAI-1 repression after an acute event such as exposure to MTS is cardio protective and may involve nonfibrinolytic functions of PAI-1. An observed increase in PAI-1 at a later stage may be a potential risk factor for developing a cardiac event.

In summary, we investigated global gene expression profiles of heart tissue of mice exposed chronically to MTS, and following smoking cessation. We demonstrate transcriptional repression and decreased protein abundance of PAI-1 in whole heart tissue of mice exposed to MTS. Importantly, the results suggest that individual susceptibility to the development of cardiac disease in smokers may depend on early events, including PAI-1 regulation. Future work will examine the manifestation of cardiac disease in PAI-1 knockout mice exposed to MTS.
